# A novel CDK4 inhibitor for myeloid protection in chemotherapy-treated triple-negative breast Cancer

**DOI:** 10.1007/s10637-025-01550-7

**Published:** 2025-06-06

**Authors:** Ava Safaroghli-azar, Laychiluh B Mekonnen, Ramin Hassankhani, Jimma Lenjisa, Sunita KC Basnet, Hajer Batayneh, Muhammed H Rahaman, Shudong Wang

**Affiliations:** Drug Discovery and Development, Clinical and Health Sciences, University of South Australia, Adelaide, South Australia 5001 Australia

**Keywords:** CDK4 inhibitor, Chemotherapy-induced myelosuppression, RB-negative tumour, Triple negative breast cancer

## Abstract

**Background:**

Despite advances in cancer treatment, chemotherapy remains a cornerstone of clinical practice. However, its efficacy is often compromised by dose-limiting haematologic toxicities. Recent strategies aim to enhance chemotherapy tolerability while preserving its effectiveness. One emerging approach involves selective CDK4 inhibitors to serve as myeloid-protective agents in retinoblastoma (RB)-negative tumours, such as triple-negative breast cancer (TNBC). Because bone marrow (BM) cells rely on RB for proliferation, CDK4 inhibitors may protect these cells while sparing RB-deficient tumour cells. The present study investigated the potential of AU2–94, a first-in-class CDK4 inhibitor, to protect BM cells during myelosuppressive chemotherapy in TNBC, beyond its established application in RB-positive cancers.

**Methods:**

This study employed in vitro, ex vivo, and in vivo experiments to evaluate the myeloid-protective effects of AU2–94 against chemotherapy-induced damage.

**Results:**

AU2–94 induced a transient G1 arrest that protects BM cells from chemotherapy-induced apoptosis by preventing DNA double-strand breaks. Pre-treatment with AU2–94 prior to 5-fluorouracil (5-FU) administration reduced BM cells apoptosis, preserved Ki67-positive cells, and mitigated declines in red blood cells and neutrophils. Similarly, AU2–94 pre-treatment before cisplatin administration reduced cisplatin-induced haematologic toxicity in RB-deficient TNBC bearing mice without compromising the efficacy of chemotherapy.

**Conclusion:**

These findings support the repurposing of AU2–94 as a myeloprotective agent, highlighting its therapeutic potential in RB-deficient tumours. With AU2–94 advancing to clinical trials, these results underscore its broader therapeutic promise, extending to both RB-positive and RB-negative cancer treatment.

**Graphical abstract:**

Schematic representation of the proposed mechanism by which AU2–94 preserves bone marrow cells from cisplatin-induced toxicity in RB-deficient cancer. (A) DNA-damaging chemotherapeutic drugs like cisplatin induce tumour cell death and provoke double-strand DNA damage in rapidly proliferating cells, including bone marrow (BM) cells, leading to unwanted myelosuppression. (B) Pre-administration of the selective CDK4 inhibitor AU2–94 before cisplatin induces transient G1 arrest in BM cells, a phase critical for monitoring DNA integrity. This intervention prevents chemotherapy-induced double-strand DNA damage in BM cells, thereby aiding in myelopreservation. Given that TNBC-derived tumour cells often carry genetic mutations in the retinoblastoma (RB) protein, these cells are resistant to CDK4 inhibition. Hence, the addition of AU2–94 to a cisplatin regimen does not interfere with treatment outcomes but solely functions as a myelopreservation strategy. 
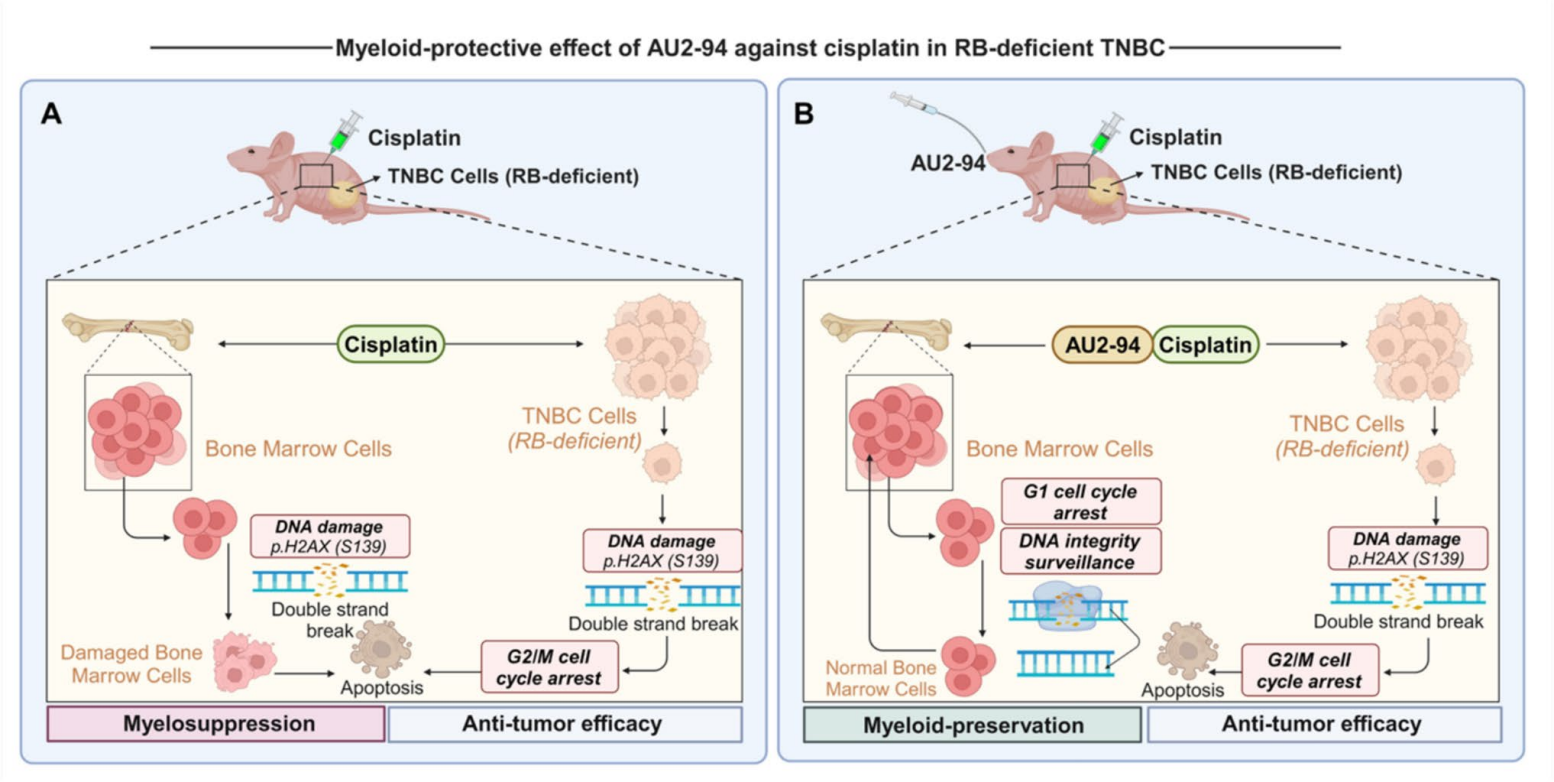

**Supplementary Information:**

The online version contains supplementary material available at 10.1007/s10637-025-01550-7.

## Introduction

Triple-negative breast cancer (TNBC) presents a significant global health challenge due to its aggressive nature, early onset, high risk of metastasis, and limited treatment options [1]. Chemotherapy remains the cornerstone of TNBC treatment [2]; however, it frequently causes severe haematologic toxicities, necessitating dose reductions and delays [3, 4]. Conventional supportive care measures, such as platelet (PLT) and red blood cell (RBC) transfusions and growth factor administration, also function as alleviative rather than prophylactic intervention and, therefore, do not prevent treatment interruptions [2]. Given these, substantial efforts have focused on directly protecting bone marrow (BM) cells—the precursors of blood cell lineages—from chemotherapy-induced damage.

Previous studies suggest that induction of transient G1 arrest in cells before chemotherapy exposure reduces their sensitivity to chemotherapy [5]. Since haematopoietic stem cells (HSPCs) rely on CDK4/6-cyclin D1 for proliferation [6], this approach may also be effective to prevent chemotherapy-induced myelosuppression. However, to preserve treatment efficacy, it is imperative that tumour cells remain unaffected by the induced G1 arrest [7]. These concepts have unveiled a new therapeutic potential for CDK4/6 inhibitors as myeloid-protective agents in retinoblastoma (RB)-deficient cancers, with trilaciclib emerging as the pioneer in this class [7, 8]. Trilaciclib has shown promising myeloid protection in small cell lung cancer (SCLC) [9]; however, its development in other cancers, particularly TNBC, faced challenges. This divergent outcome highlights the need for alternative treatments that can offer consistent protection across various cancers and different chemotherapy regimens.

Studies on HPSCs have revealed that, unlike CDK4—which only regulates cell proliferation [10, 11], CDK6 also controls cell survival and differentiation [6, 12–14]. This distinct behaviour suggests that selective CDK4 inhibition exerts a milder anti-proliferative effect on HPSC compared to dual CDK4/6 inhibition, potentially enabling faster progenitor recovery upon compound withdrawal by preserving a larger stem cell reservoir [15]. Although seems advantageous, it is important to ascertain that cell proliferation arrest induced by selective CDK4 inhibition is sufficient to induce protective effect. To address this, we used AU2–94, a first-in-class selective CDK4 inhibitor, which exhibits 140-fold greater selectivity for CDK4 over CDK6 [16]. Our previous investigation also demonstrated that AU2–94 exerted a more transient effect on HPSCs compared to the dual CDK4/6 inhibitor palbociclib, resulting in a faster recovery of hematopoietic stem cells and subsequently higher peripheral blood cell counts during continuous daily administration [16]. Building on this premise, we evaluated the myeloid-protective effect of AU2–94 in TNBC models, where nearly one-third of the tumours carry RB mutations [17]. Our findings revealed that AU2–94-induced G1 arrest protected BM cells from DNA-damaging chemotherapeutic agents. Importantly, this protection was achieved without compromising chemotherapy’s efficacy in RB-deficient MDA-MB-468 TNBC xenografts.

## Materials and methods

### Chemicals

AU2–94 was kindly provided by Changzhou LeSun Pharmacuticals Ltd., China, following previously reported synthetic procedures [18]. Cisplatin, 5-fluorouracil (5-FU), and gemcitabine were purchased from MedChemExpress (New Jersey, USA), while paclitaxel was obtained from the Royal Adelaide Hospital pharmacy (Adelaide, Australia). Primary antibodies against phospho-H2AX (Ser139), RB, CDK4, CDK6, c-Myc, cyclin D1, cyclin E1/E2, p16, CDK2, thymidylate synthase (TS), cleaved PARP (c-PARP), phospho-JNK (Thr183/Tyr185), phospho-p38/MAPK (Thr180/Tyr182), caspase-3, GAPDH and β-tubulin were purchased from Cell Signaling Technology (Victoria, Australia).

### Cell culture

Breast cancer cell lines were cultured in RPMI-1640, or Dulbecco’s Modified Eagle Medium (DMEM) supplemented with 10% fetal bovine serum (FBS) in a humidified incubator at 37 °C with 5% CO_2_. Murine BM cells were isolated from the tibiae and femurs of BALB/c mice and cultured in DMEM medium, containing 25% FBS, 5% L-glutamine (Sigma-Aldrich, Australia), and 1.5% streptomycin/penicillin solution (Sigma-Aldrich, Australia).

### MTT assay

The MTT assay was employed to assess the effects of AU2–94 on BM cells and breast cancer cells. To evaluate the myeloprotective potential of AU2–94, BM cells (10 × 10^3^) were treated with either 0.1% dimethylsulfoxide (DMSO) or AU2–94 for 24 hours, followed by exposure to cisplatin, gemcitabine, 5-FU, or paclitaxel for an additional 24 hours. Optical density (OD) was measured using a PerkinElmer plate reader (Buckinghamshire, UK), and cell viability percentages as well as GI₅₀ (Growth Inhibition 50) values were calculated using GraphPad Prism 10 (California, USA).

### Propidium iodide (PI) staining assay

Cell cycle studies were conducted on breast cancer cells and murine BM cells (10–30 × 10^4^ cells/well) following a 24-hour treatment with AU2–94. A wash-out experiment was conducted by treating BM cells with AU2–94 for 24 hours, rinsing with phosphate-buffered saline (PBS), and incubating in fresh medium for another 24 hours.

To assess the effect of AU2–94 on chemotherapy-induced cell cycle arrest, BM cells and MDA-MB-468 cells were treated with AU2–94 or 0.1% DMSO for 24 hours, followed by exposure to cisplatin or gemcitabine for an additional 24 or 48 hours. Subsequently, cells were fixed with ethanol, stained with PI solution (50 μg/ml PI, 0.1 mg/ml RNase A, 0.05% Triton X-100), and analyzed using a CytoFLEX flow cytometer (CytExpert, Beckman Coulter, California, USA).

### Caspase-3/7 activity assay

BM cells (10 × 10^3^) were seeded in a white-walled 96-well plate and treated with AU2–94 (1–4 μM) or DMSO (0.1%) for 24 hours. After removing the medium, cells were exposed to cisplatin (100 μM), 5-FU (250 μM), or paclitaxel (250 nM) for an additional 24 hours. Caspase-Glo® 3/7 reagent (Promega, Australia) was added, and luminescence was measured after a 3-hours incubation at room temperature using a PerkinElmer plate reader.

### Annexin-V/PI staining assay

BM and breast cancer cells (1–3 × 10^3^) were treated with AU2–94, and chemotherapy drugs as previously described. Following treatment, apoptosis was assessed using an Annexin-V/PI staining assay, as described in [19]. The percentage of apoptotic cells was determined using a CytoFLEX flow cytometer (CytExpert, Beckman Coulter, California, USA).

### Colony formation assay

To confirm that AU2–94 pre-treatment does not antagonize chemotherapy effects, MDA-MB-468 cells were treated with AU2–94, cisplatin, or gemcitabine for 7 days, with medium changes every 72 hours. Afterwards, cells were exposed to chemotherapy-containing media for an additional 7 days. Following treatment, colonies were stained with crystal violet solution (0.05% crystal violet, 1% formaldehyde, 1% PBS, 1% methanol). Colonies were quantified using ImageJ software (NIH, Maryland, USA).

### Western blot

BM cells or MDA-MB-468 cells treated with AU2–94 and chemotherapeutic drugs, following the mentioned schedule. Cells were lysed following treatment, and the extracted proteins were subjected to western blot analysis as described in our previous study [20]. Band intensities were quantified using ImageJ software, and the data were normalized to a housekeeping protein.

### In vivo MDA-MB-468 xenograft study

All animal experiments were conducted in compliance with institutional ethical guidelines for animal care and were approved by the University of South Australia Animal Ethics Committee (Animal Ethics Numbers: U12–23). MDA-MB-468 breast cancer cells (5 × 10^6^) suspended in a 1:1 mixture of Matrigel (Corning, Australia) and FBS-free DMEM were injected into the right flank of female nude BALB/c mice. Once the tumour size reached 80–100 mm^3^, mice were randomly divided into five groups (*n* = 5) and treated for 21 days with the following regimens: (i) vehicle (distilled water PO QW), (ii) AU2–94 (100 mg/kg PO QW), (iii) cisplatin (5 mg/kg IP QW), (iv) and (v) AU2–94 (75 mg/kg PO QW and 100 mg/kg PO QW) administered 2 hours before cisplatin.

Mice were monitored daily for any signs of toxicity and tumour volume was measured every other day. Tumour growth inhibition (TGI) was calculated as described in our previous study [19]. %T/C was calculated using the formula: %T/C = (Vt/Vc) × 100, where Vt and Vc represent the tumour volumes of the compound-treated and vehicle-treated groups, respectively, at a particular time point. Data were analyzed using GraphPad Prism version 10 for Windows (GraphPad Software, USA).

### In vivo assessment of myeloid-protective effect of AU2–94 against 5-FU

To assess the effect of AU2–94 pre-dosing on 5-FU-induced myelosuppression, female BALB/c mice were injected with EMT-6 cells (0.5 × 10^6^). Once tumours were established, mice were randomly assigned into four treatment groups (*n* = 10) and received the following treatments: (i) vehicle (distilled water PO and 0.9% saline solution, IP), (ii) a single dose of AU2–94 (200 mg/kg PO), (iii) 5-FU (100 mg/kg IP), and (iv) AU2–94 (200 mg/kg PO) administered 2 hours before 5-FU (100 mg/kg IP). To prepare each compounds for in vivo administration, AU2–94 was dissolved in distilled water. 5-FU was also dissolved in 0.9% saline solution according to previous reports [21].

Two- and six-days post-treatment, spleen and BM cells were collected from 5 mice in each treatment groups. The blood samples were randomly collected from 3 mice in each treatment group and sent to Gribbles Veterinary Pathology (Adelaide, Australia) for complete blood counts (CBC) analysis.

BM cells were isolated by flushing the femur and tibia bones with ice-cold Hank’s balanced salt solution (HBSS, Sigma-Aldrich, Australia) containing 0.5 mM ethylenediaminetetraacetic acid (EDTA, Sigma-Aldrich, Australia) and 2% FBS. Harvested cells were centrifuged, RBCs were lysed by eBioscience™ 1X RBC Lysis Buffer (Invitrogen, Australia), and single-cells were stained with Annexin-V/PI (BD Biosciences, Australia) or phycoerythrin (PE) mouse anti-Ki-67 (BD Biosciences, Australia) according to the manufacturer’s instructions. For Ki-67 staining, cells were fixed and permeabilized using BD Cytofix/Cytoperm™ Fixation/Permeabilization Solution and BD Perm/Wash™ Buffer, respectively. Cells were then exposed to PE anti-Ki-67 antibody for 1 hour at 4 °C. Flow cytometry analysis was performed using a CytoFLEX flow cytometer (CytExpert, Beckman Coulter, California, USA).

### AlphaLISA® SureFire® ultra™ assay

AlphaLISA® SureFire® Ultra™ assay was employed to assess the expression levels of RB and phosphorylated RB (serine-780) in breast cancer cell lines and BM cells, respectively. BM cells (100 × 10^3^ cells/well) were treated with AU2–94 for 24 hours. Cells were lysed using AlphaLISA Lysis Buffer (PerkinElmer, Australia) and were incubated with acceptor mix, and donor mix according to the manufacturer’s instructions. Luminescence was measured using an alpha-enabled plate reader (Victor Nivo, Multilabel plate reader).

### Statistical analysis

All in vitro studies were conducted in at least three independent experiments, unless otherwise specified. Statistical analyses were performed using t-test, one-way ANOVA or two-way ANOVA where appropriate, to determine the significance of differences among means of treatment groups. Differences between means were considered significant when p ≤ 0.05. All statistical analyses were carried out using GraphPad Prism 10 (GraphPad Software, California, USA).

## Results

### AU2–94 induced reversible G1 arrest in murine BM cells

**Fig. 1:**
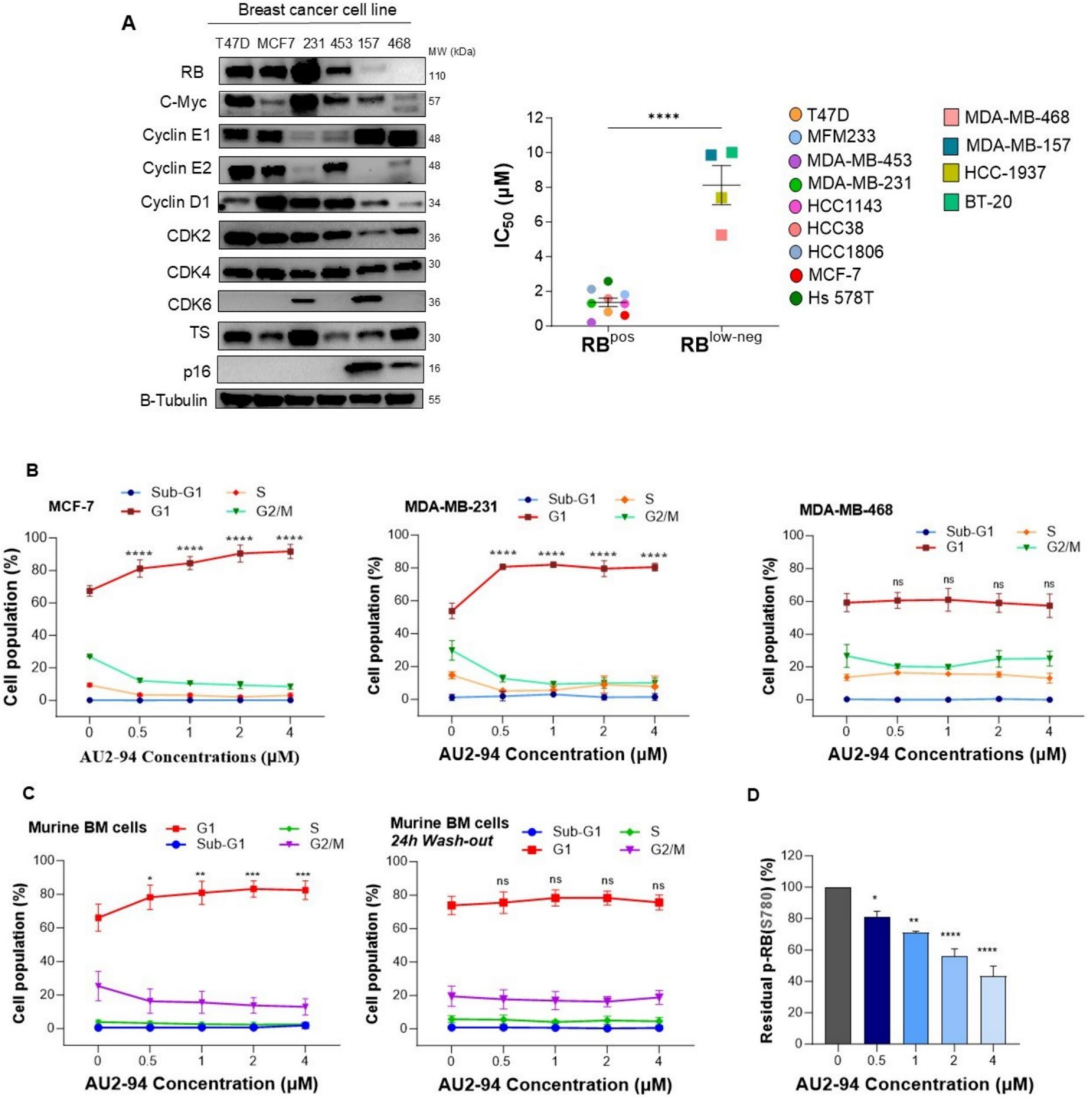
AU2–94 induced G1 arrest in RB-competent cells but not RB-deficient cells. **A** Representative western blots showing basal expression of cell cycle-related proteins in some breast cancer cell lines. Scatter plot illustrates the inverse correlation between RB expression and GI_50_ values in a panel of breast cancer cell lines. GI_50_ values were calculated as the average from three independent MTT proliferation assays conducted over 144 hours for each cell line. **B** Treatment of RB-positive cells (MCF-7 and MDA-MB-231) with AU2–94 for 24 hours resulted in G1 arrest. In contrast, AU2–94 treatment had no effect on the cell cycle distribution of RB-deficient MDA-MB-468 cells at the same time point. **C** AU2–94 induced transient G1 arrest in murine BM cells after 24 hours of treatment. **D** BM cells were exposed to increasing concentrations of AU2–94 for 24 hours, and the percentage inhibition of p-RB (S780) was assessed by alpha assay. Data are representative of three independent experiments, except for panel A, which was performed twice. Statistical significance of differences between sample groups was assessed using a t-test for panel **A**, and one-way ANOVA for the others. Error bars indicate SEM. ns, non-significant; *, *p* ≤ 0.05; **, *p* ≤ 0.01; ***, *p* ≤ 0.001; ****, *p* ≤ 0.0001 compared to control

According to previous studies, protecting BM cells from chemotherapy-induced toxicity without compromising treatment efficacy requires ensuring that AU2–94 targets RB-positive cells while sparing RB-deficient cells [7]. To evaluate this, we assessed the anti-proliferative effects of AU2–94 across a panel of breast cancer cell lines. As shown in Fig. 1A, AU2–94 effectively inhibited the proliferation of RB-expressing cells, with reduced potency in RB-deficient/low cells (MDA-MB-468, BT-20, HCC1937, and MDA-MB-157). Supplementary Fig. 1 presents RB expression levels and corresponding GI₅₀ for all cell lines. Furthermore, the AU2–94-induced G1 arrest was specific to RB-competent cells (MDA-MB-231 and MCF-7) and RB-deficient MDA-MB-468 cells remained unresponsive to the inhibitor (Fig. 1B). These results support the RB-dependent mechanism of action of AU2–94.

Building on this finding, we tested the effect of CDK4 inhibition on murine BM cells. Consistently, AU2–94 treatment (0.5–4 μM) led to a dose-dependent increase in the G1 population, which normalized within 24 hours after drug removal (Fig. 1C), indicative of a reversible effect of AU2–94. Additionally, AU2–94 treatment dose-dependently reduced RB phosphorylation (Ser-780) in BM cells (Fig. 1D), further highlighting its RB-dependent mechanism.

### AU2–94-induced G1 arrest protected BM cells from the toxicity of chemotherapeutic drugs

**Fig. 2:**
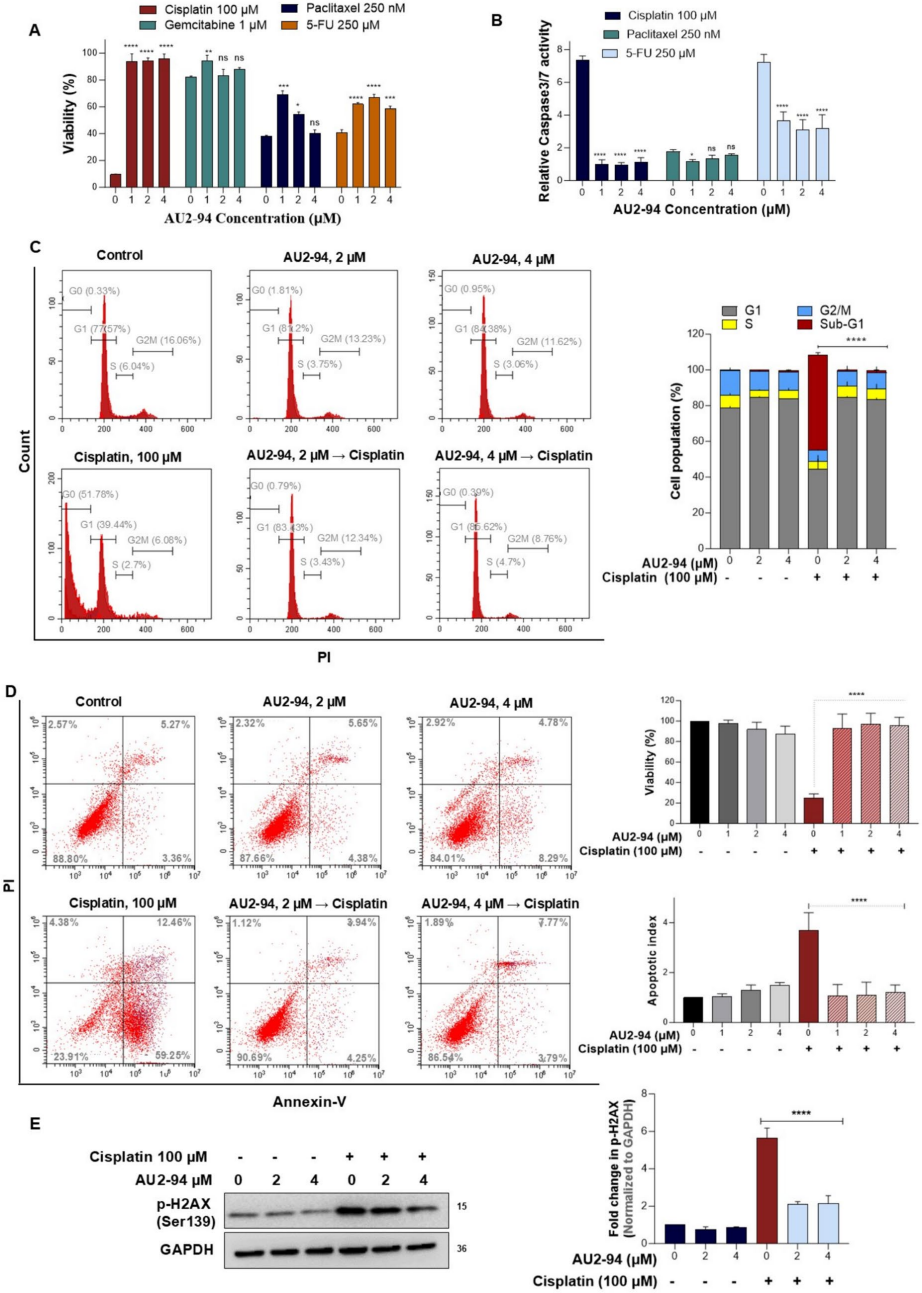
AU2–94 pre-treatment reduced the sensitivity of BM cells to chemotherapy. **A** and **B** BM cells were treated with AU2–94 or 0.1% DMSO for 24 hours and subsequently exposed to various DNA damaging chemotherapeutic drugs for another 24 hours. Cell viability was assessed using MTT assays, and the relative activity of caspase-3/7 was measured to evaluate apoptotic cell death. **C** and **D** AU2–94 pre-exposure prevented cisplatin-induced sub-G1 arrest, thereby inhibiting apoptotic cell death in BM cells. **E** Western blot analysis depicting the effect of AU2–94 pre-treatment on reducing cisplatin-induced DNA damage, as indicated by the level of γH2AX in BM cells. The fold-change in γH2AX expression was normalized to the loading control (GAPDH). Results shown are representative of three independent experiments, except for panel E, which was performed twice. Statistical significance of differences between sample groups was determined using one-way ANOVA analysis. Error bars represent SEM. ns, non-significant; *, *p* ≤ 0.05; **, *p* ≤ 0.01; ***, *p* ≤ 0.001; ****, *p* ≤ 0.0001 compared to chemotherapy-treated cells

Chemotherapeutic agents that impair DNA synthesis or disrupt mitosis, such as antimetabolites (5-FU, gemcitabine), platinum-based agents (cisplatin), and taxanes (paclitaxel), are well known for their undesired apoptotic effects on BM cells [22–25]. Accordingly, we aimed to investigate whether AU2–94-mediated G1 arrest could mitigate this cytotoxic effect on BM cells. BM cells were pre-treated with AU2–94 for 24 hours before exposure to cisplatin, gemcitabine, 5-FU, and paclitaxel for an additional 24 hours. As expected, AU2–94 pretreatment prevented the toxic effect of chemotherapeutic drugs on BM cells, as evidenced by the higher viability of pre-treated cells and the lower caspase-3/7 activity than chemotherapy-treated cells (Fig. 2A and B).To confirm that the protective effect was mediated by AU2–94-induced G1 arrest, we focused on cisplatin, as a myelosuppressive agent in TNBC treatment [26]. Before exposure to high-dose cisplatin (100 μM), BM cells were pre-exposed to AU2–94 for 24 hours at the concentrations of 2 μM and 4 μM, which induced the highest G1 arrest according to our results. As shown in Fig. 2C, cisplatin alone increased sub-G1 population, whereas AU2–94 pre-treatment sustained cells at G1 and prevented their transition to sub-G1 following cisplatin exposure.

Studies suggest that G1 phase accumulation reduces sensitivity to DNA-damaging agents by preventing apoptosis associated with E2F1 activation or allowing time for DNA repair [5]. Consistent with this, our results demonstrated that AU2–94 pre-treatment significantly reduced apoptosis following cisplatin exposure (Fig. 2D) and decreased phosphorylation of histone H2AX (Ser-139), known as γH2AX (Fig. 2E). Since a reduction in γH2AX suggests a potential decrease in double-strand DNA breaks [27], these findings propose that AU2–94 protects BM cells from cisplatin-induced apoptosis, at least in part, by mitigating DNA damage.

### AU2–94 pre-treatment protected BM cells from the apoptotic effect of 5-FU in vivo

To assess the translational potential of AU2–94 in vivo, we evaluated its early protective effects against chemotherapy-induced myelosuppression. Chemotherapeutic agents used in breast cancer, particularly platinum-based drugs, often cause early-onset myelosuppression, with blood cell count reaching their nadir 7–14 days post-treatment [22]. Given that many chemotherapy regimens are administered on a weekly basis, these effects can be cumulative, thereby increasing the risk of severe haematologic toxicity with subsequent drug administration [22]. Therefore, early myelo-protection is critical to mitigate cumulative toxicity.

**Fig. 3:**
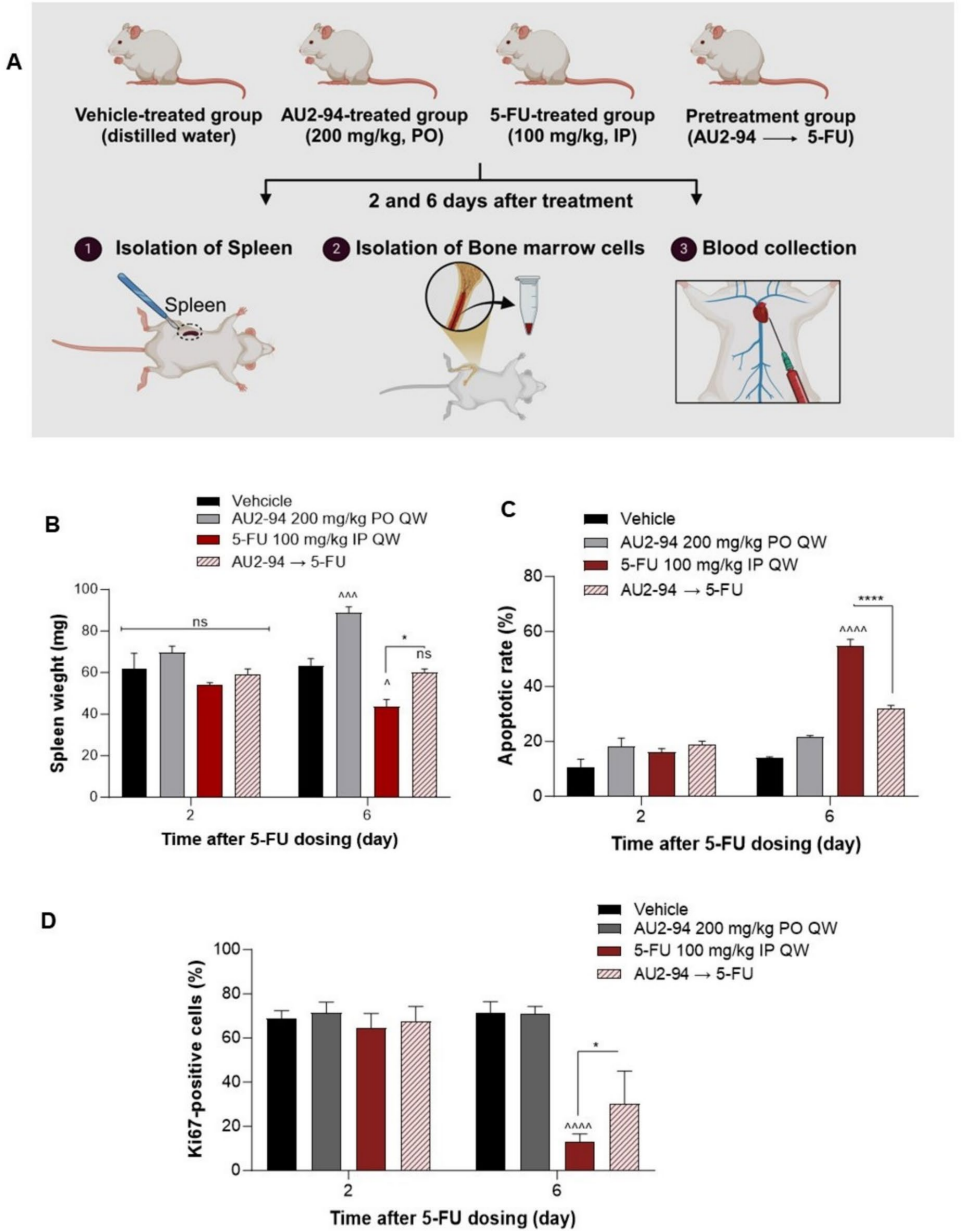

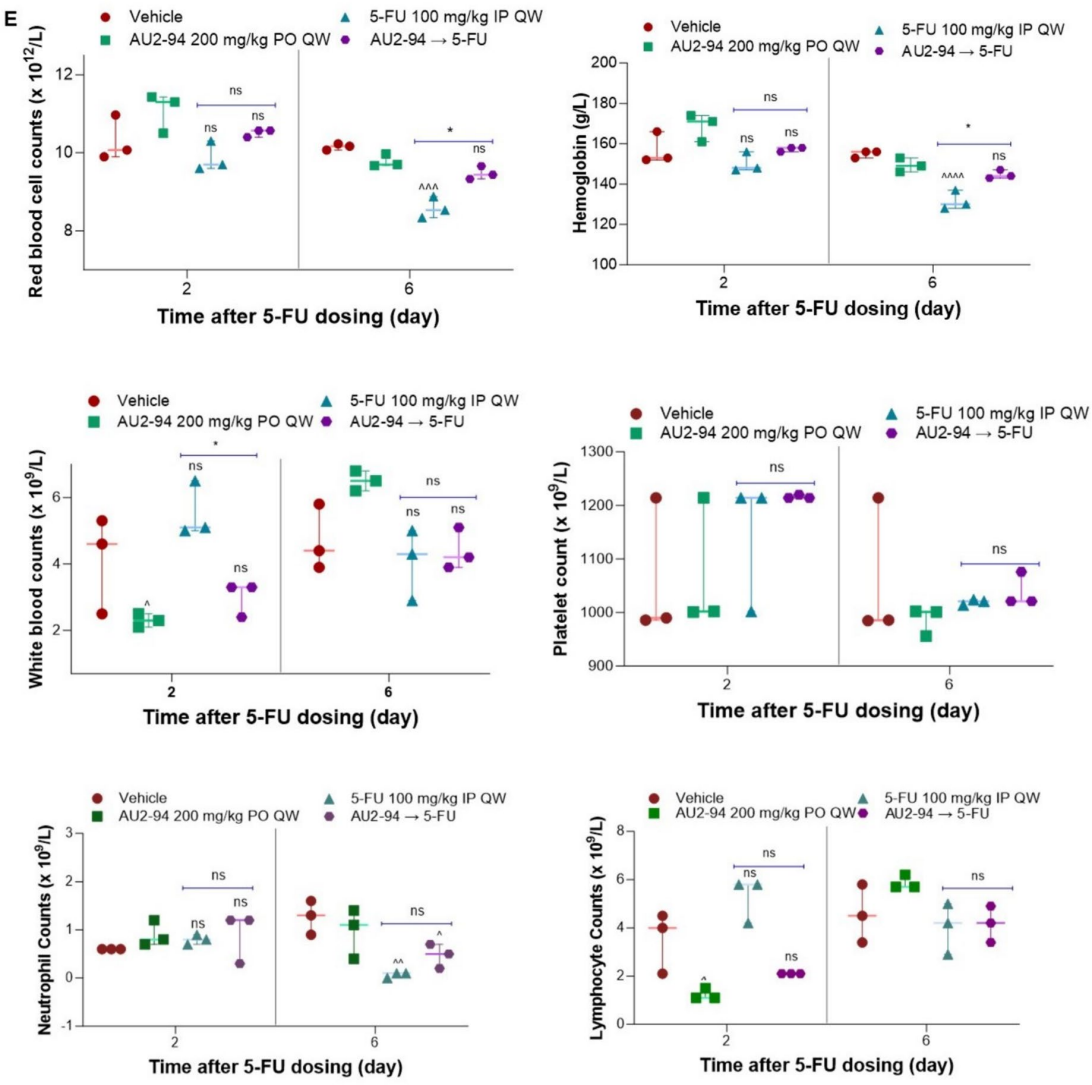
AU2–94 pre-treatment mitigated the myelosuppressive effect of 5-FU in murine BM cells. **A** Experimental procedure illustrating treatment of female BALB/c mice (*n* = 10 per group) with vehicle (distilled water, PO), a single dose of AU2–94 (200 mg/kg, PO), 5-FU (100 mg/kg, IP), or AU2–94 (200 mg/kg, PO) administered 2 hours prior to 5-FU injection. Spleens, BM cells, and blood samples were collected at 2- and 6-days post-5-FU administration. **B** Spleen weight differences among treatment groups post-5-FU treatment. **C** Comparison of apoptotic events in BM cells among treatment groups, assessed by Annexin-V/PI staining. **D** Percentage of Ki-67 positive cells in BM among treatment groups, determined by Ki-67 intracellular staining. **E** Blood samples collected from 3 mice per group at 2 and 6 days after treatment. Statistical significance of differences between sample groups was evaluated using two-way ANOVA in GraphPad Prism 10. Error bars represent SEM. ns, non-significant; *, *p* ≤ 0.05; **, *p* ≤ 0.01; ***, *p* ≤ 0.001; ****, *p* ≤ 0.0001 compared to the 5-FU treated group. ^, *p* ≤ 0.05; ^^, *p* ≤ 0.01; ^^^, *p* ≤ 0.001; ^^^^, *p* ≤ 0.0001 compared to the vehicle-treated group

In this study, we used a single dose of 5-FU (100 mg/kg), previously shown to induce acute myelosuppression in mice [28, 29], to establish a model with acute myelosuppression. Moreover, to better mimic the cancer setting, we inoculated breast cancer–derived EMT-6 cells into BALB/c mice to evaluate the early haematologic protective effect of AU2–94. In this study, mice were treated with vehicle, a single dose of AU2–94 (200 mg/kg, PO), a single dose of 5-FU (100 mg/kg, IP), or AU2–94 administered 2 hours before 5-FU injection, based on the compound’s time to reach maximum plasma concentrations (Tmax). Spleen, BM cells, and blood samples were collected 2- and 6-days post-treatment (Fig. 3A). Given the short duration of treatment (6 days), no significant differences in tumour size were observed between the groups. Our findings demonstrated that AU2–94 pre-treatment mitigated 5-FU-induced haematopoietic toxicity, as evidenced by its protective effects on the spleen, a surrogate organ of haematopoiesis, and BM cells. As depicted in Fig. 3B, while 5-FU treatment significantly reduced spleen weight by day 6, AU2–94 pre-administration prevented this decline, maintaining spleen weight at levels comparable to the vehicle group (Fig. 3B). Moreover, AU2–94 significantly reduced 5-FU-induced apoptosis in BM cells. Analysing the apoptosis induction in BM cells 6 days after 5-FU administration showed that 58% of BM cells underwent apoptosis, whereas this amount reduced to 27.6% in the pre-treated group (Fig. 3C and supplementary Fig. 2A and B). Additionally, Ki67 staining revealed a pronounced decline in the population of Ki67-positive cells 6 days after 5-FU administration. Although AU2–94 did not fully prevent this loss, the percentage of Ki67-positive cells were significantly higher in the pre-treated group compared to 5-FU alone on day 6 (Fig. 3D, supplementary Fig. 3A and B). Given that dividing BM cells are particularly vulnerable to chemotherapy-induced toxicity [7, 30], these findings suggest that AU2–94’s ability to reduce 5-FU-induced apoptosis may preserve the population of proliferative BM cells, potentially facilitating haematopoietic recovery.

Concurrently with the protection in BM cells, we observed an improvement in peripheral blood cell counts in pre-treatment group. While we failed to find significant changes in peripheral blood cell count 2 days after dosing due to early time point, we found that 5-FU treatment reduced RBCs, Hb levels, and neutrophils on day 6 (Fig. 3E). Notably, AU2–94 pre-treatment resulted in higher RBC counts and Hb levels compared 5-FU single agent (Fig. 3E). Furthermore, despite a decrease in neutrophils, AU2–94 pre-treatment maintained higher neutrophil levels compared to 5-FU-treated mice (Fig. 3E). The only transient effect observed with AU2–94 treatment was a temporary reduction in lymphocyte count, detected two days after compound administration, attributed to lymphocyte sensitivity to CDK4 inhibition [31]. This reduction returned to baseline levels by day 6.

### AU2–94 pre-treatment did not antagonize chemotherapy in RB-deficient MDA-MB-468 cells

**Fig. 4:**
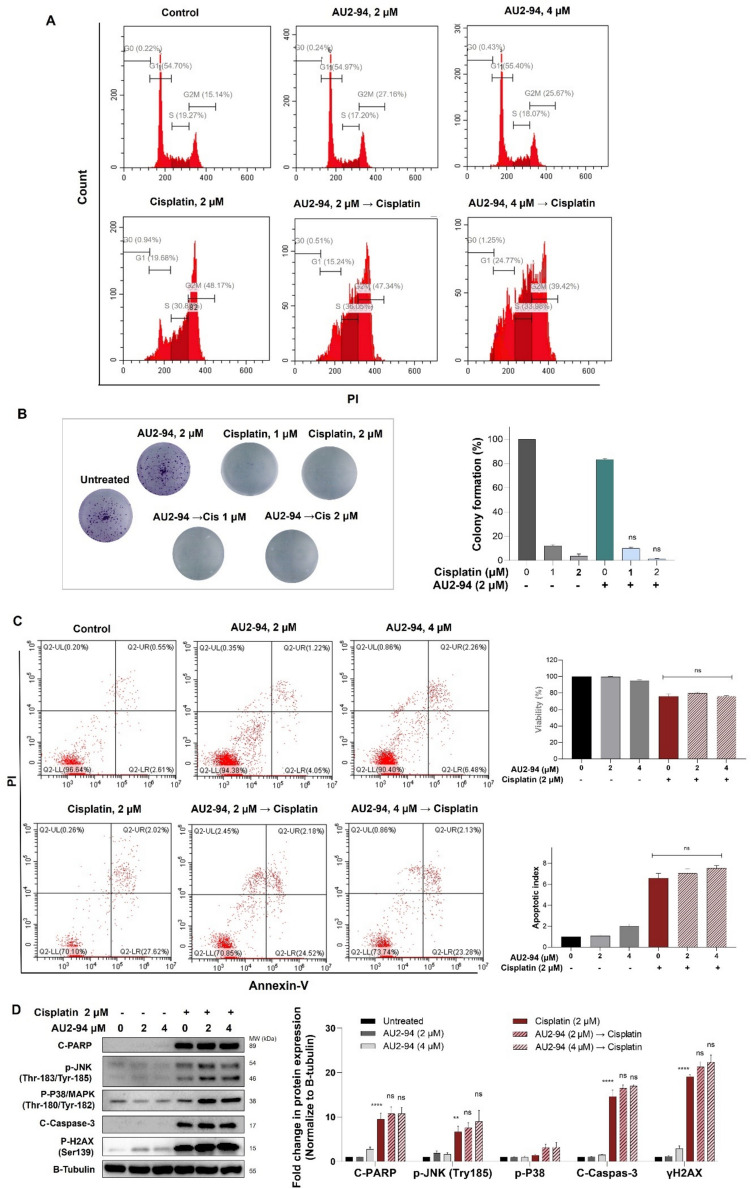
The impact of AU2–94 pre-treatment on cisplatin-induced anti-cancer effect in RB-deficient MDA-MB-468 cells. **A** Flow cytometric analysis of cell cycle distribution in MDA-MB-468 cells following 24-hour pre-exposure to AU2–94 (2 and 4 μM) followed by 48-hour incubation with cisplatin (2 μM). **B** Colony formation assay in MDA-MB-468 cells treated with AU2–94 for 7 days, followed by exposure to cisplatin for another 7 days. Colonies were quantified using ImageJ software. **C** Assessment of apoptosis induction in MDA-MB-468 cells treated with AU2–94, cisplatin, and AU2–94 pre-treatment. **D** Immunoblotting analysis of MDA-MB-468 cells exposed to indicated concentrations of AU2–94 and cisplatin, showing protein expression levels. Results are representative of three independent experiments. Statistical significance between treatment groups was determined using one-way ANOVA analysis. Error bars represent SEM. ns, non-significant compared to cisplatin-treated cells; **, *p* ≤ 0.01; ****, *p* ≤ 0.0001 compared to control. Cis: Cisplatin

Having established the myelo-protective effect of AU2–94 pretreatment, it was crucial to ensure that this treatment does not compromise chemotherapy efficacy in RB-deficient MDA-MB-468 TNBC cells. Our results showed that 48 hours treatment with cisplatin (2 μM) induced G2/M arrest and apoptosis in MDA-MB-468 cells. Notably, 24-hour pre-treatment with AU2–94 did not interfere with this anti-cancer properties, as evidenced by the sustained G2/M cell cycle arrest (Fig. 4A), inhibition of colony formation (Fig. 4B), and induction of apoptosis (Fig. 4C). Moreover, cisplatin, whether administered alone or following AU2–94 pre-treatment, consistently increased the phosphorylation of H2AX (Ser-139) and JNK (Tyr-185), as well as the cleavage of PARP and caspase-3 in MDA-MB-468 cells (Fig. 4D).

To further confirm that CDK4 inhibition in RB-deficient cells does not antagonize chemotherapy drugs, we also exposed AU2–94 pre-treated cells to gemcitabine. Similarly, AU2–94 pre-treatment did not interfere with gemcitabine-induced effects on cell cycle, apoptosis, and protein expression (Supplementary Fig. 4).

### AU2–94 pre-treatment mitigated cisplatin toxicity without compromising efficacy in an RB-deficient TNBC xenograft model

**Fig. 5:**
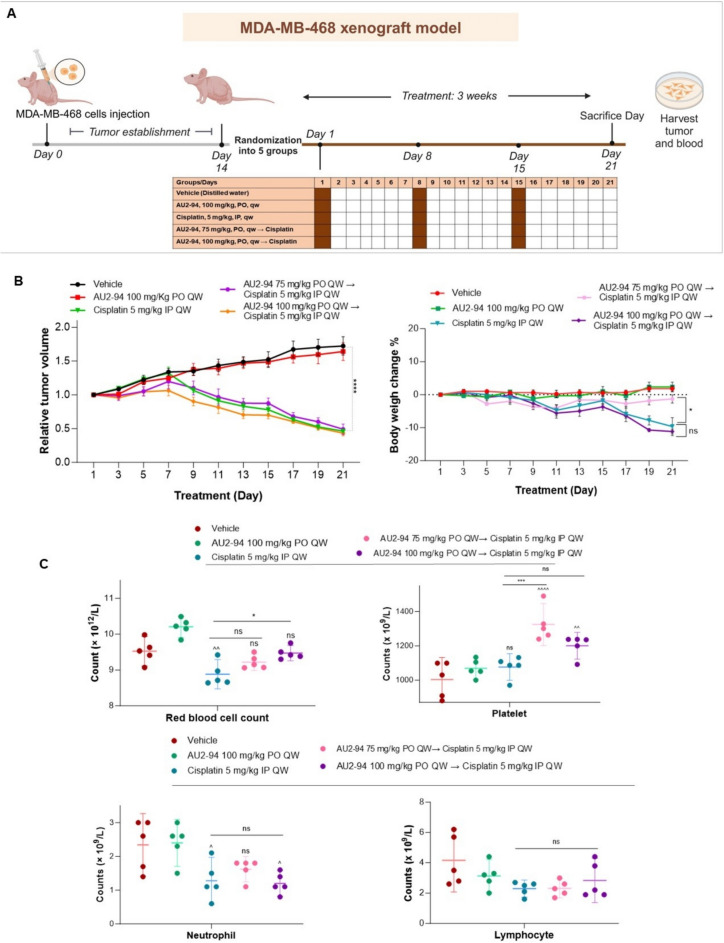
The effect of pre-administration of AU2–94 before cisplatin in the MDA-MB-468 xenograft model. **A** and **B** Treatment schedule and relative changes in tumour volume and weight over a 21-day treatment cycle in MDA-MB-468-bearing BALB/c nude mice. Treatments included vehicle (distilled water PO QW), AU2–94 (100 mg/kg PO QW), cisplatin (5 mg/kg IP QW), AU2–94 (75 mg/kg PO QW) administered 2 hours before cisplatin, and AU2–94 (100 mg/kg PO QW) administered 2 hours before cisplatin. **C** Evaluation of peripheral blood cell counts 21 days after treatment initiation, illustrating that pre-administration of AU2–94 at 75 mg/kg before each cisplatin injection protected against chemotherapy-induced haematologic toxicity. Statistical significance between treatment groups was assessed using two-way ANOVA analysis. Error bars represent SEM. ns, non-significant; *, *p* ≤ 0.05; **, *p* ≤ 0.01; ***, *p* ≤ 0.001; ****, *p* ≤ 0.0001 compared to cisplatin-treated group. ^, *p* ≤ 0.05; ^^, *p* ≤ 0.01; ^^^^, *p* ≤ 0.0001 compared to vehicle group

To further explore the effect of AU2–94 pre-treatment on cisplatin-induced anticancer efficacy in vivo, MDA-MB-468-bearing BALB/c nude mice were treated weekly with vehicle, AU2–94 (100 mg/kg, PO), cisplatin (5 mg/kg, IP), or AU2–94 (75/100 mg/kg, PO) prior to cisplatin administration for 21 days (Fig. 5A). Unlike AU2–94, which did not alter tumour volume, cisplatin significantly reduced tumour size, resulting in a T/C = 28.19% on day 21. Importantly, AU2–94 pre-treatment did not affect cisplatin’s efficacy, as indicated by T/C = 27.02% and 28.75% for AU2–94 at doses of 75 mg/kg and 100 mg/kg, respectively (Fig. 5B). Moreover, while mice in the vehicle and AU2–94 (100 mg/kg) monotherapy groups maintained stable body weight, those treated with cisplatin (5 mg/kg) experienced wight loss. Notably, although pre-treatment with the higher dose of AU2–94 (100 mg/kg) did not prevent cisplatin-induced weight loss, pre-administration of the lower dose of AU2–94 (75 mg/kg) prevented this effect, with a statistically significant difference observed by day 21 (Fig. 5B). We next assessed the effect of AU2–94 pre-treatment on peripheral blood cells. Pre-treatment with AU2–94 (75 and 100 mg/kg) increased RBC count compared to cisplatin alone (Fig. 5C). Moreover, although cisplatin did not reduce PLT counts, AU2–94 pre-treatment, especially at 75 mg/kg, increased PLT levels (Fig. 5C), which despite being notably elevated, remained within the normal reference range (908–1792 × 10^9^/L), thereby mitigating concerns about thrombosis. Besides RBCs and PLT, we also evaluated the effect of AU2–94 pre-treatment on neutrophils, which differentiate from myeloid progenitors and are critical components of innate immunity. Since myeloid progenitors are sensitive to chemotherapy-induced toxicity [32], their preservation by AU2–94 was critical. We found that pre-treatment with AU2–94 at 75 mg/kg, but not at 100 mg/kg, partially preserved this cell population, suggesting a potential protective trend despite the absence of statistical significance. Lymphocyte counts remained comparable across all treatment groups (Fig. 5C).

## Discussion

The concept of myeloid protection through CDK4/6 inhibition holds significant importance in cancer therapy, as chemotherapy remains a cornerstone in the treatment of many cancers, and its myelosuppressive effects often compromise therapeutic outcomes [8]. In the present study, we showed that selective CDK4 inhibition in BM cells using AU2–94 induced G1 arrest, thereby protecting these cells from chemotherapy-induced apoptosis—likely by preventing DNA double-strand breaks. In vivo findings also demonstrated that AU2–94 pre-treatment before 5-FU dosing significantly reduced apoptosis in BM cells, preserved the population of Ki67-positive cells, and mitigated the early decline in blood cell counts. These myeloid-protective effects are consistent with previous reports on trilaciclib in SCLC [7, 30], suggesting that CDK4 inhibition alone is sufficient to accelerate haematologic recovery following chemotherapy, achieving comparable outcomes to dual CDK4/6 inhibitors.

Effective myeloid protection in clinical settings requires two key criteria. The inhibitor must induce transient G1 arrest that sufficiently protects BM cells during chemotherapy exposure, without exacerbating chemotherapy-induced myelosuppression, and it must be well-tolerated while preserving the treatment efficacy [30]. While trilaciclib has fulfilled these criteria in SCLC [9], it did not demonstrate haematologic benefit in TNBC (NCT05112536; NCT04799249), and its pre-administration compromised treatment efficacy in colorectal cancer (NCT04607668). These findings suggest that trilaciclib’s effectiveness is restricted to specific chemotherapy regimen and tumour type [33]. Given these, we sought to investigate whether AU2–94 could provide both safety and efficacy in TNBC, particularly in combination with cisplatin.

Interestingly, AU2–94 pre-administration was well-tolerated in the MDA-MB-468 xenograft model, preserved cisplatin-induced anti-cancer effects, and mitigated haematologic toxicity—most notably by protecting RBCs and, to a lesser extent, neutrophils. This pre-treatment also elevated platelet counts, suggesting a potential protection of megakaryocytic progenitors. The differential response observed between two doses of this agent could be due to the distinct sensitivities of haematopoietic lineages to the inhibitor. Previous investigation has shown early HPSCs, particularly myeloid progenitors, are more sensitive to CDK4/6 inhibition compared to other progenitors, thereby, undergoing more prolonged G1 arrest [30]. While this event may be beneficial for protection, it could necessitate a longer period for neutrophils to differentiate from the preserved progenitor cells. This factor could potentially account for the diminished protective effect of the 100 mg/kg AU2–94 dose on neutrophils, despite its beneficial impact on RBCs. Collectively, our results indicate that the 75 mg/kg dose of AU2–94 produced consistent, albeit partial, improvements in RBC and neutrophil counts, was associated with less weight loss compared to cisplatin alone, and did not compromise cisplatin’s anti-tumour efficacy. These improvements indicate a reduced likelihood of dose-limiting toxicity, potentially allowing for sustained chemotherapy exposure and improved therapeutic outcomes. Given these, administration of AU2–94 in a pre-chemotherapy dosing schedule, similar to what had been designed for trilaciclib [8, 33], could transiently arrest BM cells in G1 phase at the time of chemotherapy exposure, which ultimately protect these cells from chemotherapy-induced toxicity. Unlike continuous administration, this pre-chemotherapy dosing schedule would ensure that HPSCs resume proliferation after chemotherapy clearance, leading the haematologic benefit.

## Conclusion

Overall, our findings support the myeloid-protective potential of AU2–94 in RB-deficient TNBC model in a pre-chemotherapy dosing schedule. However, to fully elucidate the protective effect of AU2–94, further studies are warranted, particularly those involving the mechanistic basis for the dose-dependent response observed with different haematologic lineages. Future investigations should explore a broader range of AU2–94 doses to determine the relation between dose and lineage sensitivities, as well as to identify the optimal dosing that could produce maximal protective effects across various haematologic lineages. Our study was also conducted using immunodeficient mouse models, which inherently limit the ability to evaluate effects on the adaptive immune cells. Given the known immunomodulatory properties of CDK4/6 inhibitors, additional studies in immunocompetent models are conducting to assess the impact of AU2–94 on immune cell populations and its additional influence on anti-tumour immunity. With AU2–94 advancing toward its first human clinical trial, these findings would underscore its promise for broader therapeutic potential—extending beyond traditional use in RB-proficient cancers to RB-deficient malignancies.

## Supplementary Information

ESM 1 (DOCX 2552 kb)

## Data Availability

No datasets were generated or analysed during the current study.
