# Supplementary material for: A novel CDK4 inhibitor for myeloid protection in chemotherapy-treated triple-negative breast Cancer
Source: Invest New Drugs. 2025 Jun 6;43(3):728–41. doi: 10.1007/s10637-025-01550-7 (PMC12310753; doi:10.1007/s10637-025-01550-7)
Supplement: Supplementary file 1 — (DOCX 2552 kb) [file 10637_2025_1550_MOESM1_ESM.docx]

**A Novel CDK4 Inhibitor for Myeloid Protection in Chemotherapy-Treated Triple-Negative Breast Cancer**

Ava Safaroghli-azar, Laychiluh Bantie, Ramin Hassankhani, Jimma Lenjisa, Sunita KC Basnet, Hajer Batayneh, Muhammed Rahman, Shudong Wang*

Drug Discovery and Development, Clinical and Health Sciences, University of South Australia, Adelaide, SA 5000, Australia

***Correspondence:** Professor Shudong Wang, Drug Discovery and Development, Clinical and Health Sciences, University of South Australia, Adelaide, South Australia 5001, Australia. Phone: +61 (0)400266639, Email: [shudong.wang@unisa.edu.au](mailto:shudong.wang@unisa.edu.au)

**Running title:** Myeloid-protective effect of AU2-94 in TNBC treatment.

**Keywords:** CDK4 inhibitor, chemotherapy-induced myelosuppression, Rb-negative breast cancer.

**
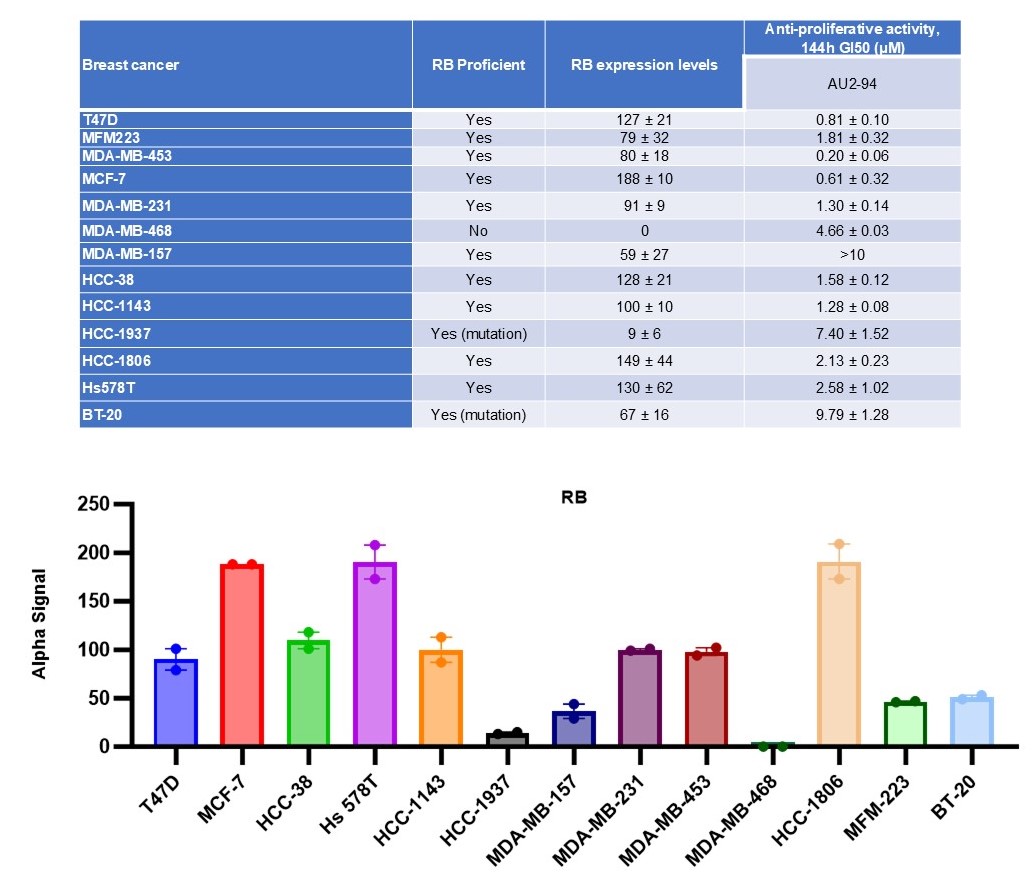
**

**Supplementary Fig. 1: Correlation between AU2-94-induced anti-proliferative effects and RB expression in breast cancer cells.** Cells were treated with AU2-94 for 144 hours, and GI₅₀ values were calculated as the average of three independent MTT proliferation assays. To assess basal RB protein expression levels, cell lysates were prepared using AlphaLISA Lysis Buffer (PerkinElmer, Australia), and RB levels was quantified using the AlphaLISA™ *SureFire*® *Ultra*™ total Rb assay (Cat# ALSU-TRB-A-HV, PerkinElmer). Luminescence was measured using an Alpha-enabled plate reader (Victor Nivo Multilabel Plate Reader). RB expression levels for all cells were normalized with housekeeping protein cofilin. The RB expression levels reported in the table are the averages of the duplicates from each experiment. The graph is representative of two independent experiments with duplicate samples.


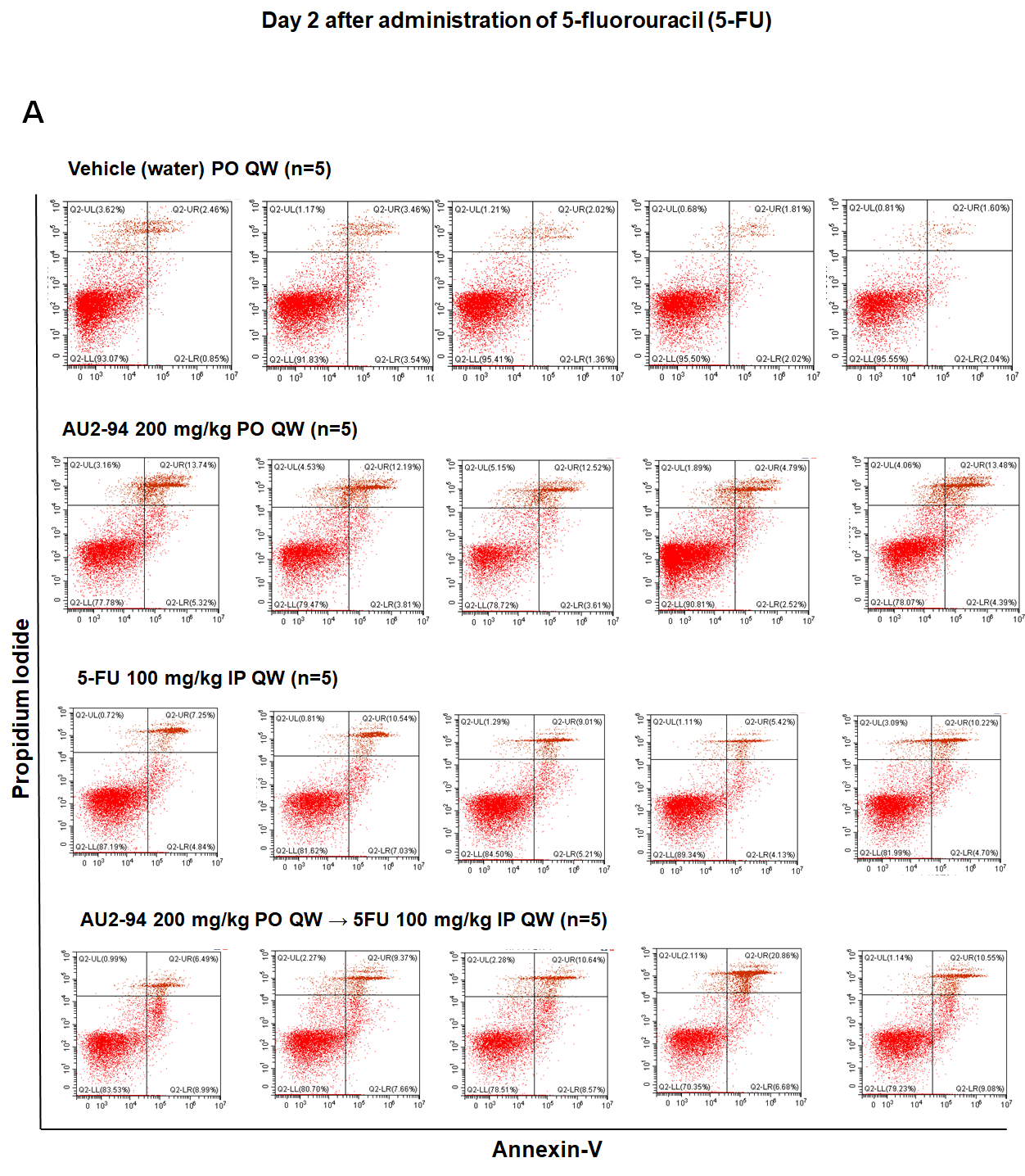


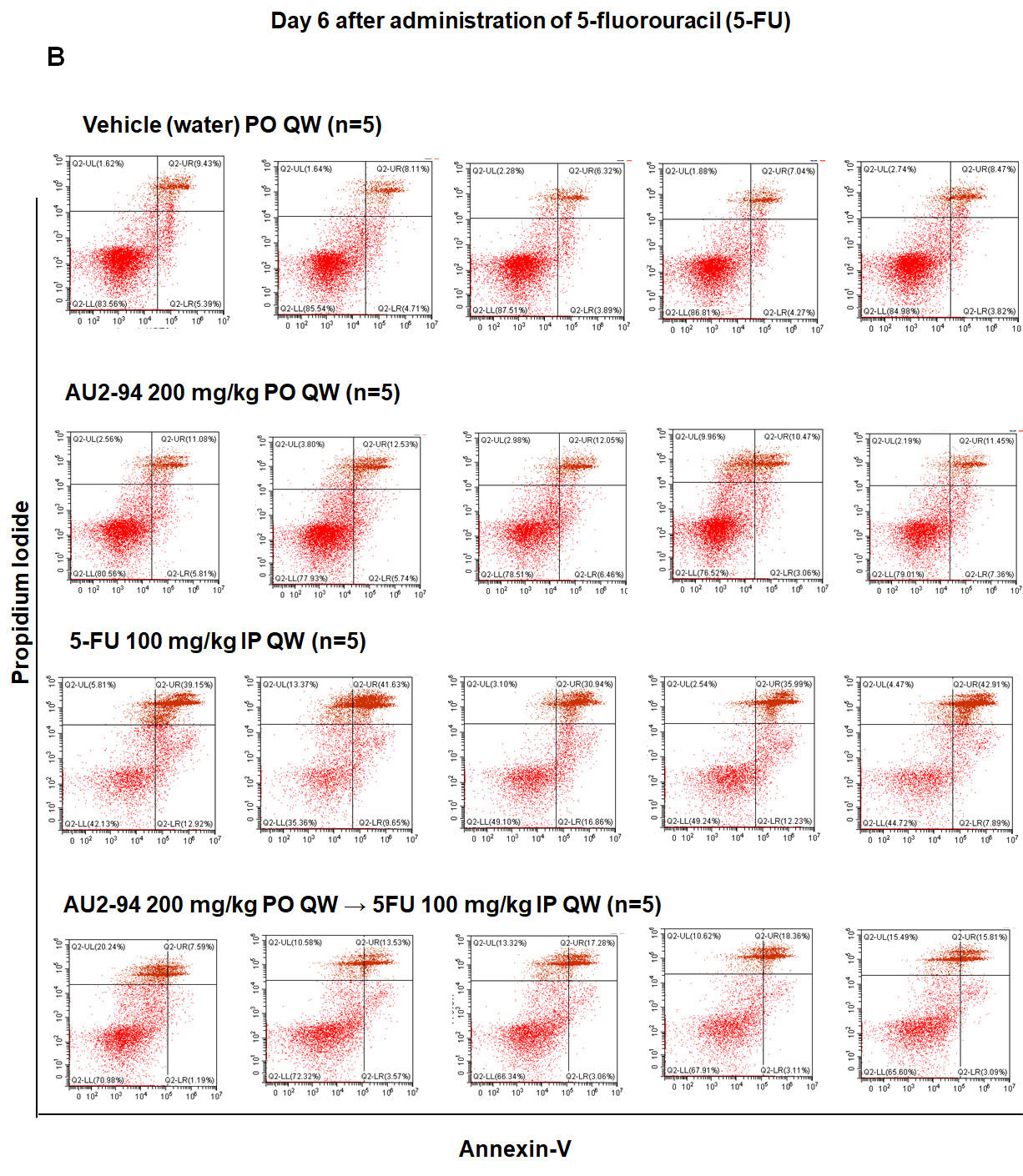


**Supplementary Fig. 2A-B: AU2-94 pre-treatment mitigates the myelosuppressive effect of 5-FU in murine BM cells.**Apoptotic events were compared in mice treated with (i) vehicle (distilled water PO QW), (ii) AU2-94 (200 mg/kg PO QW), (iii) 5-FU (100 mg/kg IP QW), or (iv) AU2-94 (200 mg/kg PO QW) administered 2 hours before 5-FU (100 mg/kg IP QW). Mice (n = 5) were euthanized 2- and 6-days post-treatment, and bone marrow (BM) cells were harvested by flushing tibia and femur bones with HBSS supplemented with FBS and EDTA. Harvested BM cells were stained with Annexin-V and PI (BD Biosciences, Australia) for 15 minutes, and apoptosis induction was evaluated using a CytoFLEX flow cytometer (CytExpert, Beckman Coulter, California, USA).


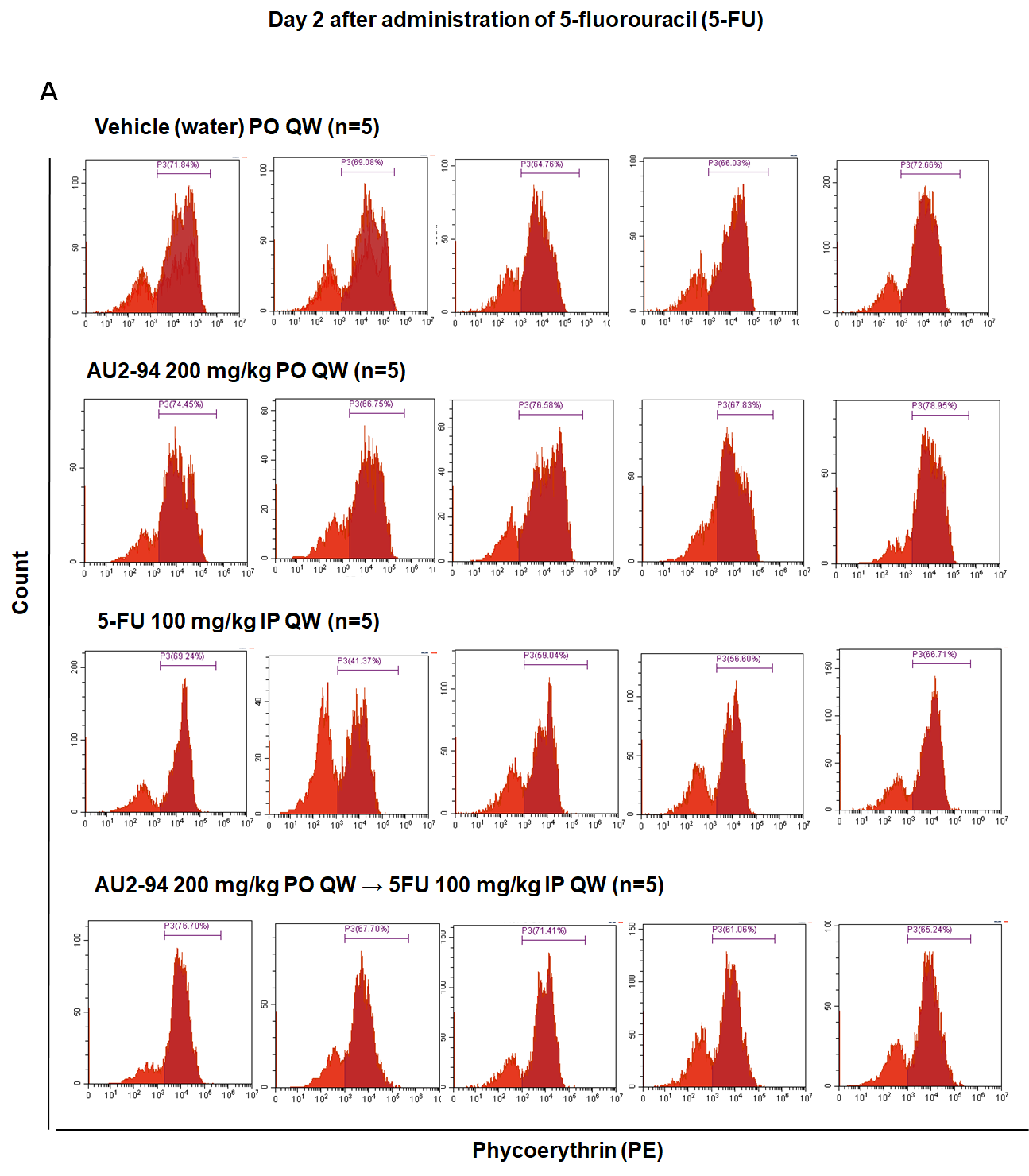


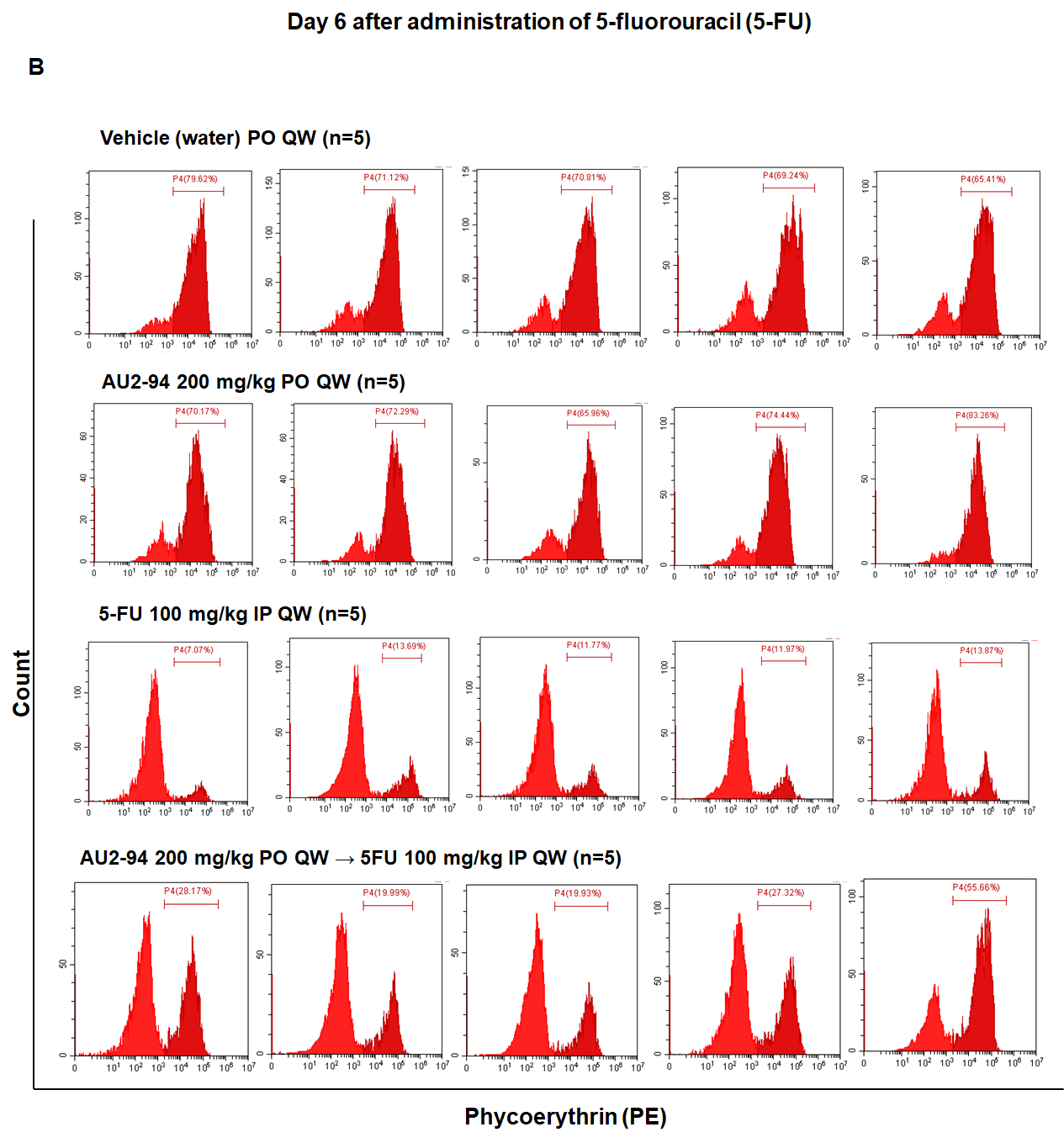


**Supplementary Fig. 3A-B: AU2-94 pre-treatment mitigates the myelosuppressive effect of 5-FU in murine BM cells.**The percentage of Ki67-positive cells were compared in mice treated with (i) vehicle (distilled water PO QW), (ii) AU2-94 (200 mg/kg PO QW), (iii) 5-FU (100 mg/kg IP QW), or (iv) AU2-94 (200 mg/kg PO QW) administered 2 hours before 5-FU (100 mg/kg IP QW). Mice (n = 5) were euthanized 2- and 6-days post-treatment, and bone marrow (BM) cells were harvested by flushing tibia and femur bones with HBSS supplemented with FBS and EDTA. Harvested BM cells were stained with phycoerythrin (PE) mouse anti-Ki-67 (BD Biosciences, Australia) for 1h at 4 ºC, and subjected to CytoFLEX flow cytometer (CytExpert, Beckman Coulter, California, USA). P4 is representative of the population of Ki-67 positive cells.


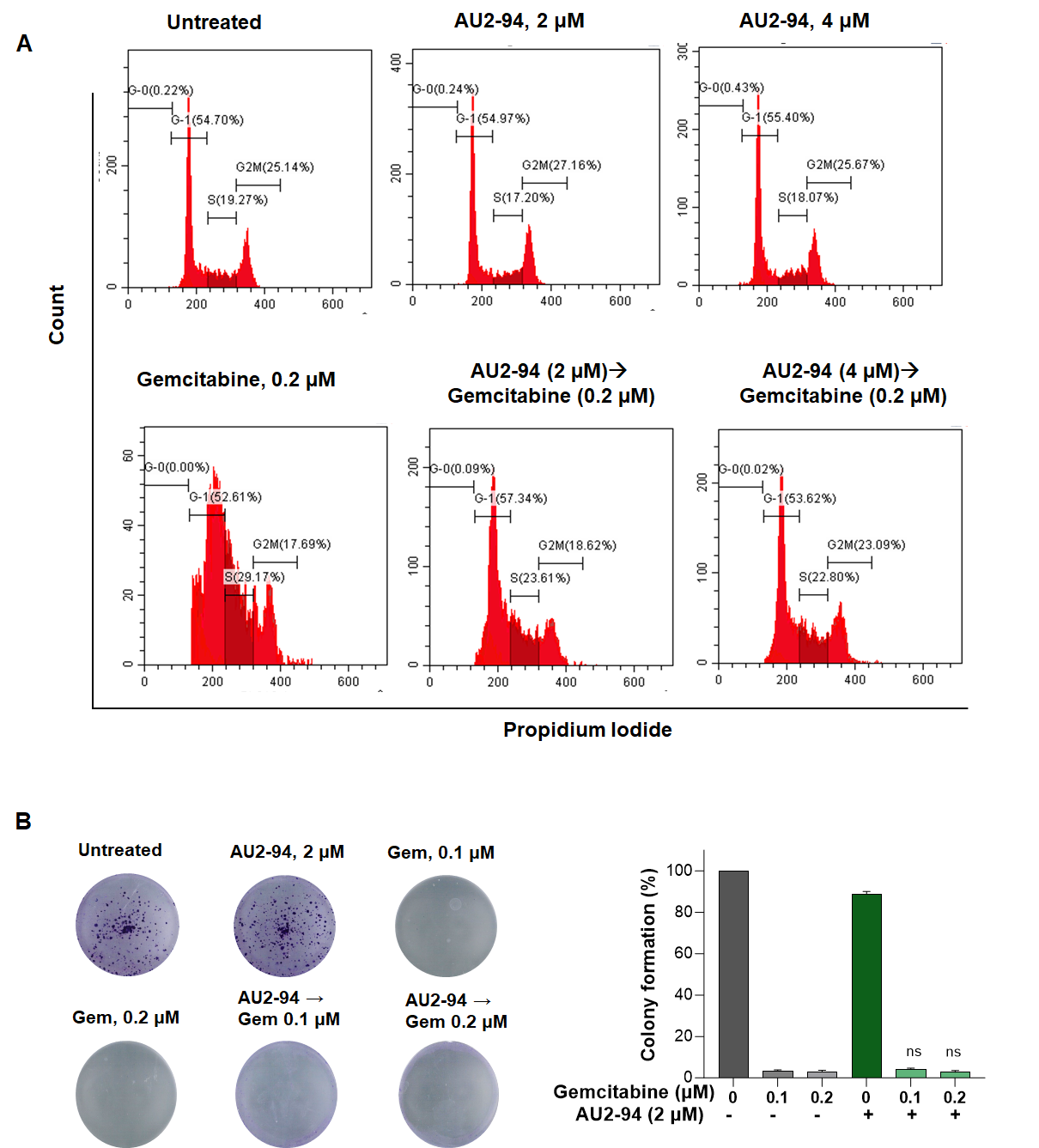


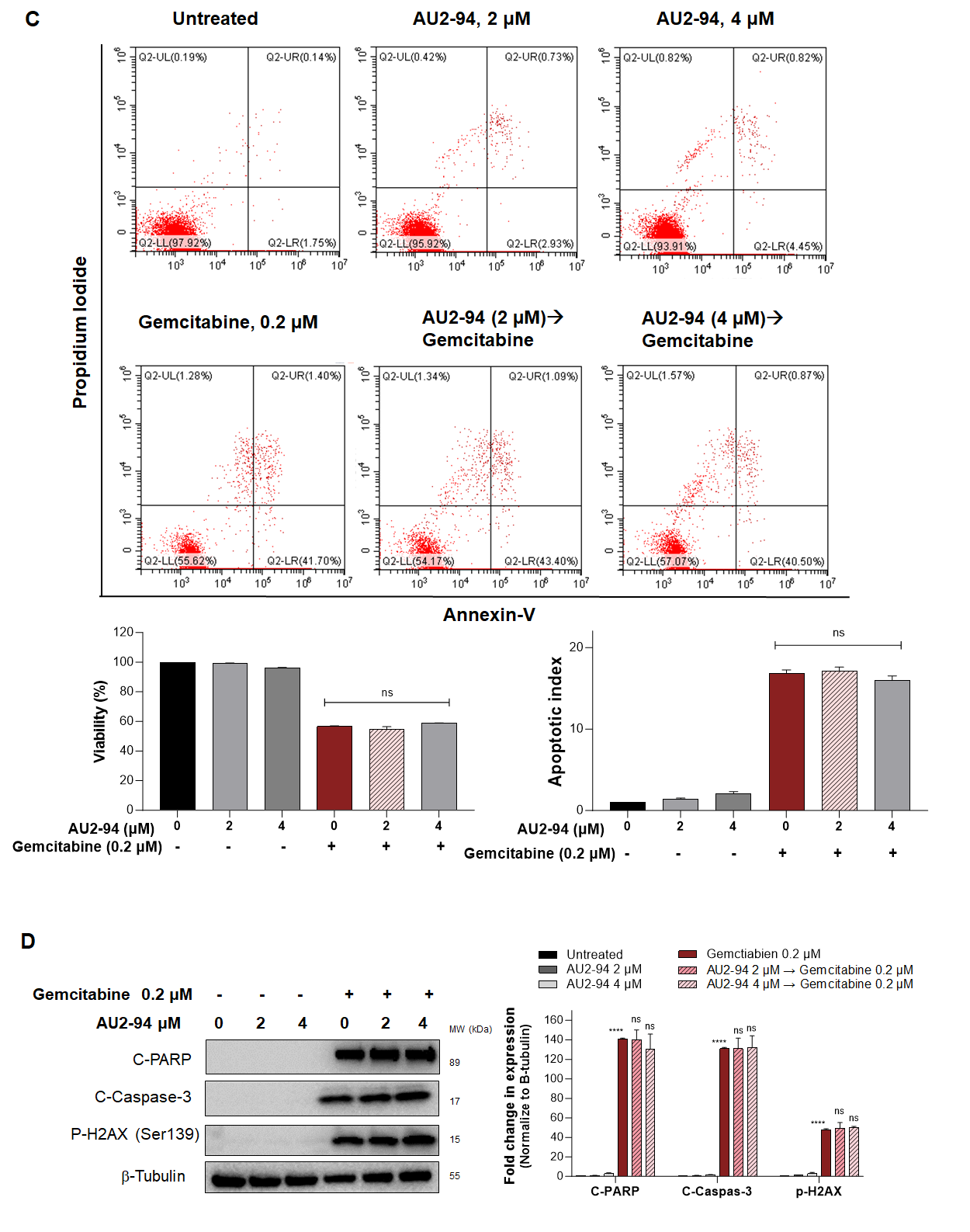


**Supplementary Fig. 4 The impact of AU2-94 pre-treatment on gemcitabine-induced anti-cancer effect in MDA-MB-468 cells.** (**A**) PI staining to evaluate cell cycle distribution. (**B**) Colony formation assay to measure long-term survival and proliferation. (**C**) Annexin-V/PI staining to assess the incidence of apoptotic cell death. (**D**) Western blot analysis of proteins associated with cell survival and apoptosis. Results are representative of three independent experiments. Statistical significance between treatment groups was determined using one-way ANOVA analysis. Error bars indicate SEM. Non-significant differences compared to gemcitabine-treated cells are denoted as "ns", while **** indicates p ≤ 0.0001 compared to control. "Gem" denotes gemcitabine.
